# Supplementary material for: Splice-Junction-Based Mapping of Alternative Isoforms in the Human Proteome
Source: Cell Rep. Author manuscript; Available in PMC 2020 Jan 15. (PMC6961840; doi:10.1016/j.celrep.2019.11.026)
Supplement: 2 [file NIHMS1546469-supplement-2.zip › DF1/PXD010154/Esophagus/Esophagus_17_PALLD_FKEDLLNNGQPEYK.pdf]

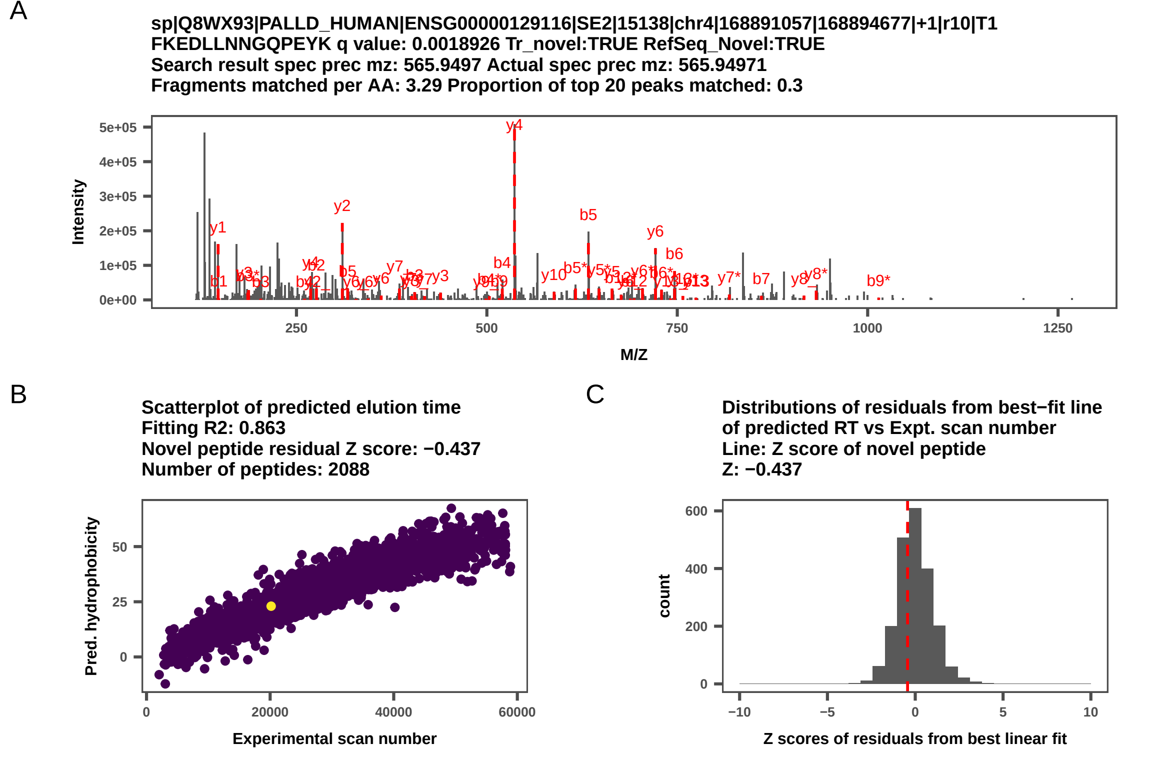
A
sp|Q8WX93|PALLD_HUMAN|ENSG00000129116|SE2|15138|chr4|168891057|168894677|+1|r10|T1
FKEDLLNNGQPEYK q value: 0.0018926 Tr_novel:TRUE RefSeq_Novel:TRUE
Search result spec prec mz: 565.9497 Actual spec prec mz: 565.94971
Fragments matched per AA: 3.29 Proportion of top 20 peaks matched: 0.3
Intensity
5e+05
4e+05
3e+05
2e+05
1e+05
0e+00
y4
y2
b5
y1
y6
b6
b4
y10
b7
b9*
250
500
750
1000
1250
M/Z
B
Scatterplot of predicted elution time
Fitting R2: 0.863
Novel peptide residual Z score: −0.437
Number of peptides: 2088
Pred. hydrophobicity
50
25
0
0
20000
40000
60000
Experimental scan number
C
Distributions of residuals from best−fit line
of predicted RT vs Expt. scan number
Line: Z score of novel peptide
Z: −0.437
count
600
400
200
0
−10
−5
0
5
10
Z scores of residuals from best linear fit
